# Complete mitochondrial genome of the western painted turtle (*Chrysemys picta bellii*, Testudines: Emydidae) in Korea

**DOI:** 10.1080/23802359.2023.2288439

**Published:** 2023-12-04

**Authors:** Jaehong Park, Seung-Min Park, Jae-Hyuk Choi, Ha-Cheol Sung, Dong-Hyun Lee

**Affiliations:** a School of Biological Sciences and Biotechnology Graduate School, Chonnam National University, Gwangju, Korea; b Research Center of Ecomimetics, Chonnam National University, Gwangju, Korea; c Department of Biological Sciences, College of Natural Sciences, Chonnam National University, Gwangju, Korea

**Keywords:** *Chrysemys picta bellii*, Emydidae, mitochondrial genome

## Abstract

The complete mitochondrial genome of *Chrysemys picta bellii* in Korea was sequenced and characterized. The mitochondrial genome consists of 37 genes (13 protein-coding genes, 22 transfer RNA genes, and 2 ribosomal RNA genes) and a noncoding region. Phylogenetic analysis based on the mitochondrial genome sequences revealed that *C. p. bellii* from Korea formed a cluster with *C. p. bellii* from China and *C. picta* from the USA, while showing clear separation from other turtle species within the *C. picta* cluster. This study presented the first complete mitochondrial genome from *C. p. bellii* in Korea, offering crucial information for managing invasive species and protecting the local ecosystem.

## Introduction

**Figure 1.**
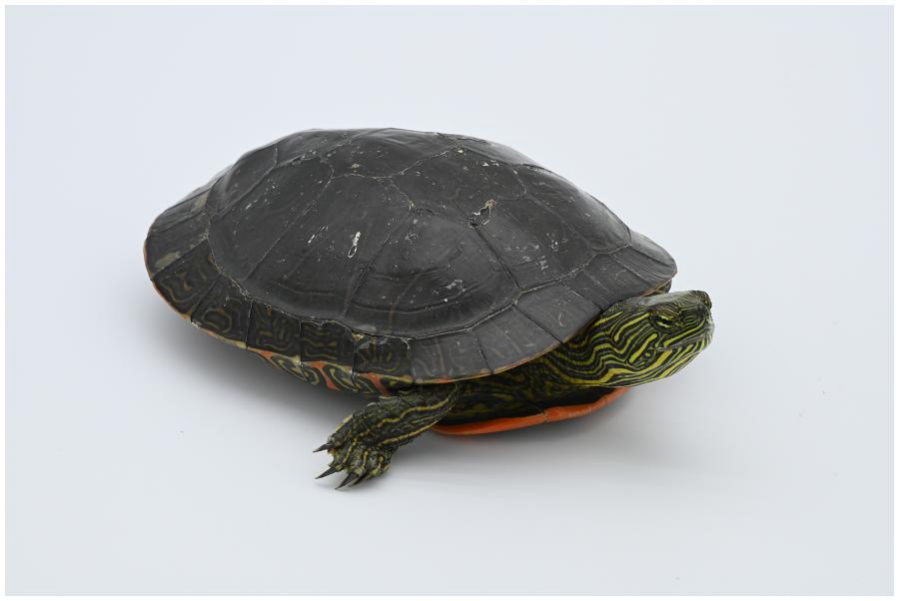
The image of *Chrysemyc picta bellii*. This picture was taken by Seung-Min Park.

The western Painted Turtle, *Chrysemys picta bellii* (Testudines: Emydidae; Schneider 1783) is a subspecies of *C. picta* native to the western areas along the Pacific coast of the United States (Van, 2011) (Figure 1). Painted turtles are popular pets because of their distinctive belly patterns and leg markings (Ernst and Lovich 2009). However, the global pet trade has led to an increase in the release of reptiles into the wild (Sung and Fong 2018; Marshall et al. 2020; Oh and Hong 2007), causing disruptions in ecosystems and biodiversity (Ham et al. 2022; Park et al. 2022; Cheon et al. 2023). *C. p. bellii* has also been reported in the wild in Korea (Park et al. 2020), necessitating management strategies. Despite this, only a little information is available about the mitochondrial genome of *C. p. bellii*. This study aims to sequence and analyze the complete mitochondrial genome of *C. p. bellii* found in South Korea, contributing to phylogenetic research and the management of invasive species.

## Materials and methods

The *C. p. bellii* specimen was captured from Gwangju (35°7'38.24"N, 126°52'14.27"E), Korea, and the tail tissue was acquired while the specimen was in a live state. This research received official sanction from Yeongsangang River Basin Environmental Office, a division of the Korea Ministry of Environment (permission number: 2021-8). Total genomic DNA was extracted from the tail tissue using the DNeasy Blood & Tissue kit (Qiagen, Valencia, CA). The extracted DNA sample was deposited at the Museum of Wildlife, located in Research Center of Ecomimetics, Chonnam National University, Korea (https://biology.jnu.ac.kr; Ha-Cheol Sung; shcol2002@jnu.ac.kr) under species voucher number 2023-RCE-CPB001. The mitochondrial genome was analyzed using Illumina NovaSeq X plus platform (Illumina, San Diego, CA), which was performed by Macrogen (Seoul, Korea). Raw sequence data were checked using FastQC, and adaptor trimming and quality filtering were conducted with Trimmomatic (Andrews 2010; Bolger et al. 2014). Subsequently, *de novo* assembly was performed using SPAdes 3.15.0, and the filtered reads were aligned using BLAST (Bankevich et al. 2012; Altschul et al. 1990). Finally, the complete sequence was annotated using MITOS2 web server (Bernt et al. 2013).

To investigate the phylogenetic position of *C. p. bellii*, the complete mitochondrial genome sequences of 16 species in *Testudines* were extracted from GenBank, and the phylogenetic tree was constructed using MEGA X software (Kumar et al. 2018). Specifically, the sequences were aligned using MUSCLE algorithm and the phylogenetic tree was made using maximum likelihood method and GTR + G model with 1,000 bootstrap replicates (Waddell and Steel 1997; Edgar 2004). GTR + G substitution model was selected as the best model by MEGA X. Also, Bayesian inference analysis was performed using MrBayes 3.2.7a (Ronquist et al. 2012). Markov Chain Monte Carlo (MCMC) chains were executed for 20,000,000 generations, with sampling every 1,000 steps and an initial 25% burn-in process. The final average standard deviation of split frequencies was 0.0065.

## Results

**Figure 2.**
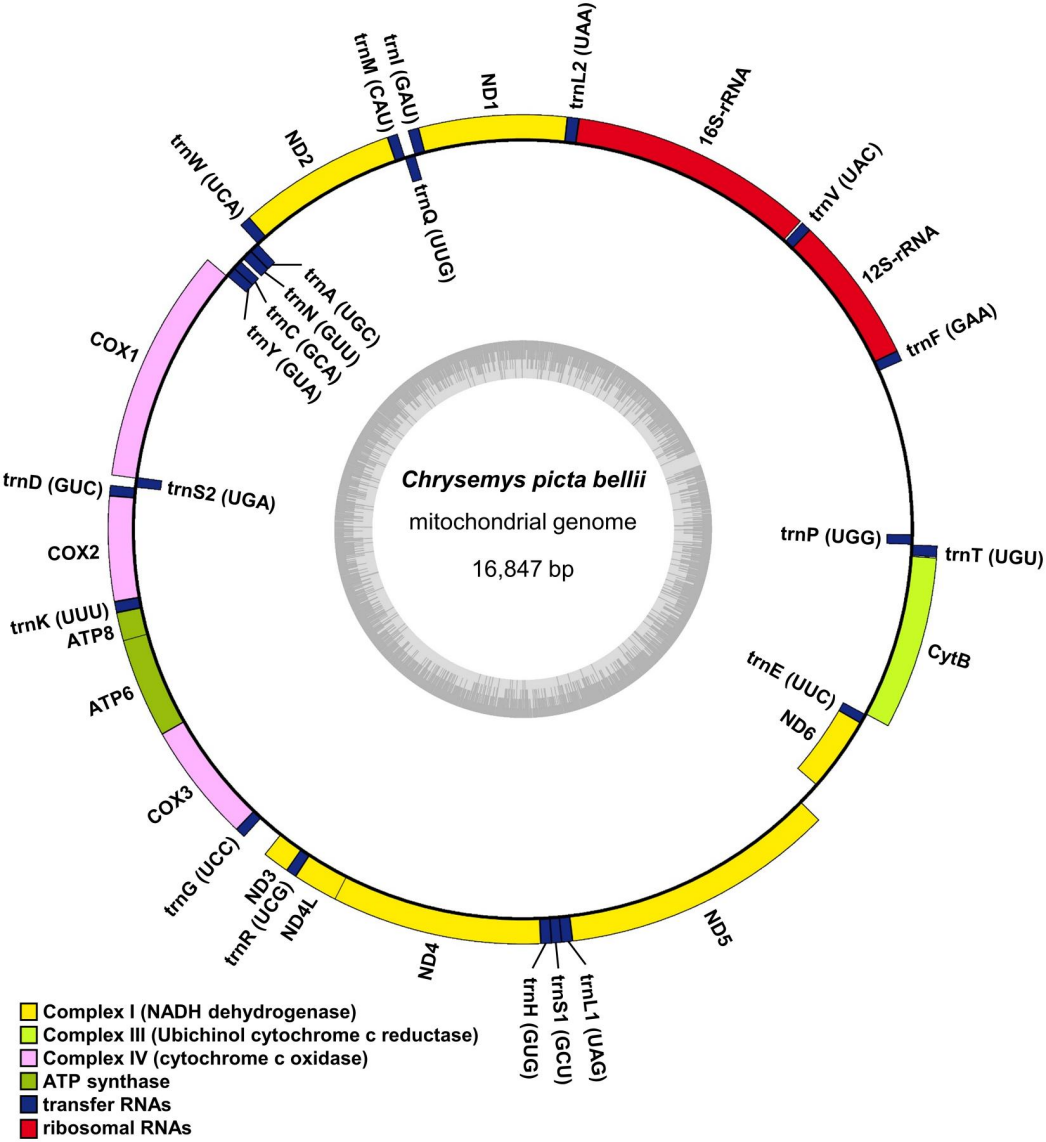
Mitochondrial genome map of *Chrysemys picta bellii*. Genes encoded on the heavy strand are written outside the circle and genes on the light strand inside the circle.

The complete mitochondrial genome of *C. p. bellii* is 16,874 bp in length and has been deposited in GenBank (Accession number: OR253893). It contained 13 protein-coding genes (PCGs), 22 transfer RNA (tRNA) genes, 2 ribosomal RNA (rRNA) genes, and a putative long non-coding control region. Among these, 12 PCGs, 14 tRNA genes, and 2 rRNA genes were encoded in the heavy strand, whereas 1 PCG (NADH dehydrogenase subunit 6) and 8 tRNA genes were encoded in the light strand (Figure 2). Of 13 PCGs, 11 PCGs have canonical mitochondria start codons, including AUG for COX2, ATP8, ATP6, COX3, ND4L, ND5, ND6, and Cytb, and AUA for ND1, ND2, and ND3. COX1 and ND4 have alternative initiation codon (GUG). Also, COX3 and ND4 have an incomplete stop codon ending with UA and U, respectively.

The nucleotide composition of the *C. p. bellii* mitochondrial genome was as follows: *A* = 34.4%, *T* = 26.7%, *G* = 12.8%, and *C* = 26.0%. It is similar to that of *C. p. bellii* from China (KF874616; *A* = 34.4%, *T* = 26.6%, *G* = 13.0%, and *C* = 26.0%) and *C. picta* from the USA (AF069423; *A* = 34.4%, *T* = 26.8%, *G* = 12.8%, and *C* = 25.9%). The sequence of *C. p. bellii* from Korea shows a high similarity of 99% with *C. p. bellii* from China and *C. picta* from the USA (99%) but less similar with *Trachemys scripta elegans* from Korea (MW019443; 90%), *Mauremys reevesii* from Korea (FJ469674; 81%), or *Pelodiscus sinensis* from Korea (AY962573; 75%).

**Figure 3.**
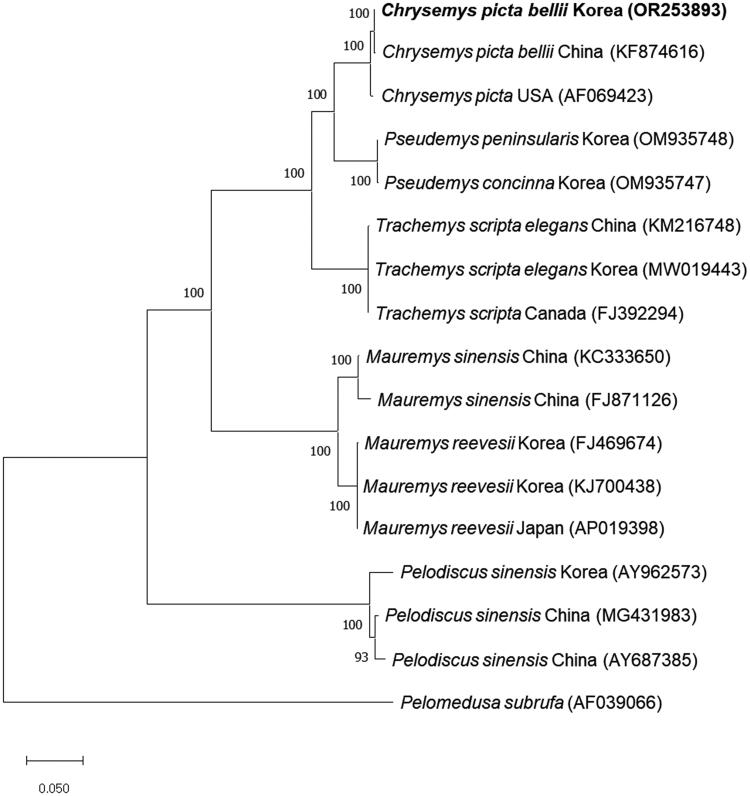
Phylogenetic tree of *C. picta bellii* and other related species based on mitochondrial genome sequences. Phylogenetic analysis was performed using MEGA X software. GenBank accession numbers of each mt genome sequence are given in the bracket after the species name, and the bootstrap value based on 1,000 replicates is represented on each node. *Pelomedusa subrufa* was used as an outgroup to root the tree.

To investigate the phylogenetic relationship between *C. p. bellii* from Korea and other turtles, we constructed the phylogenetic tree using the complete mitochondrial genome sequence of *C. p. bellii* from Korea and the sequences from the other 16 species. Consistent with sequence identity, the phylogenetic tree showed that *C. p. bellii* from Korea was clustered closely with *C. p. bellii* from China and *C. picta* from the USA and completely separated from other turtles including *T. s. elegans*, *M. reevesii*, and *P. sinensis* (Figure 3). We also obtained a similar result from Bayesian inference analysis (Figure S2).

## Discussion and conclusion

We identified the complete mitochondrial genome of *C. p. bellii* from Korea and elucidated the phylogenetic relationship with other turtles by constructing a phylogenetic tree. In the phylogenetic tree, *C. p. bellii* from Korea is placed closer to other *C. picta* than remaining turtles including *Pseudemys concinna*, *T. s. elegans*, and *M. reevesii*. Nonetheless, in *C. picta* cluster, *C. p. bellii* from Korea is separated from one from China and *C. picta* from the USA with a high bootstrap score.

Many countries have suffered from problems with invasive species, and non-native turtle *C. p. bellii* was found in the wild of Korea. This means that *C. p. bellii* could also have a negative effect on the ecosystem of Korea. However, there is only a little information about the complete mitochondrial genome of *C. p. bellii*. These data can contribute to further studies on biodiversity and management of *C. p. bellii* which is a non-native species in many countries including Korea.

## Supplementary Material

Supplemental Material

## Data Availability

GenBank accession number from the complete mitochondrial genome of *Chrysemys picta bellii* (OR253893) has been registered with the NCBI database (https://www.ncbi.nlm.nih.gov/OR253893). The associated BioProject, BioSample, and SRA accession numbers are PRJNA993820, SAMN36409323, and SRR25242840, respectively.
