# Supplementary material for: Complete mitochondrial genome of the western painted turtle (Chrysemys picta bellii, Testudines: Emydidae) in Korea
Source: Mitochondrial DNA B Resour. 2023 Dec 4;8(12):1316–9. doi: 10.1080/23802359.2023.2288439 (PMC10776060; doi:10.1080/23802359.2023.2288439)
Supplement: Supplemental Material [file TMDN_A_2288439_SM5905.docx]

**Supplementary materials**

**Complete mitochondrial genome of the western painted turtle (*Chrysemys picta bellii*, Testudines: Emydidae) in Korea.**

Jaehong Park^a^*, Seung-Min Park^a^*, Jae-Hyuk Choi^a^, Ha-Cheol Sung^b,c^, and Dong-Hyun Lee^b,c^

^a^School of Biological Sciences and Biotechnology Graduate School, Chonnam National University, Gwangju, Korea; ^b^Research Center of Ecomimetics, Chonnam National University, Gwangju, Korea; ^c^Department of Biological Sciences, College of Natural Sciences, Chonnam National University, Gwangju, Korea

*These authors contributed equally to this work.

**CONTACT**

Ha-Cheol Sung (shcol2002@jnu.ac.kr); Dong-Hyun Lee (donghyunlee73@jnu.ac.kr); Department of Biological Sciences, College of Natural Sciences, Chonnam National University, 77, Yongbong-ro, Buk-gu, Gwangju, 61186, Korea


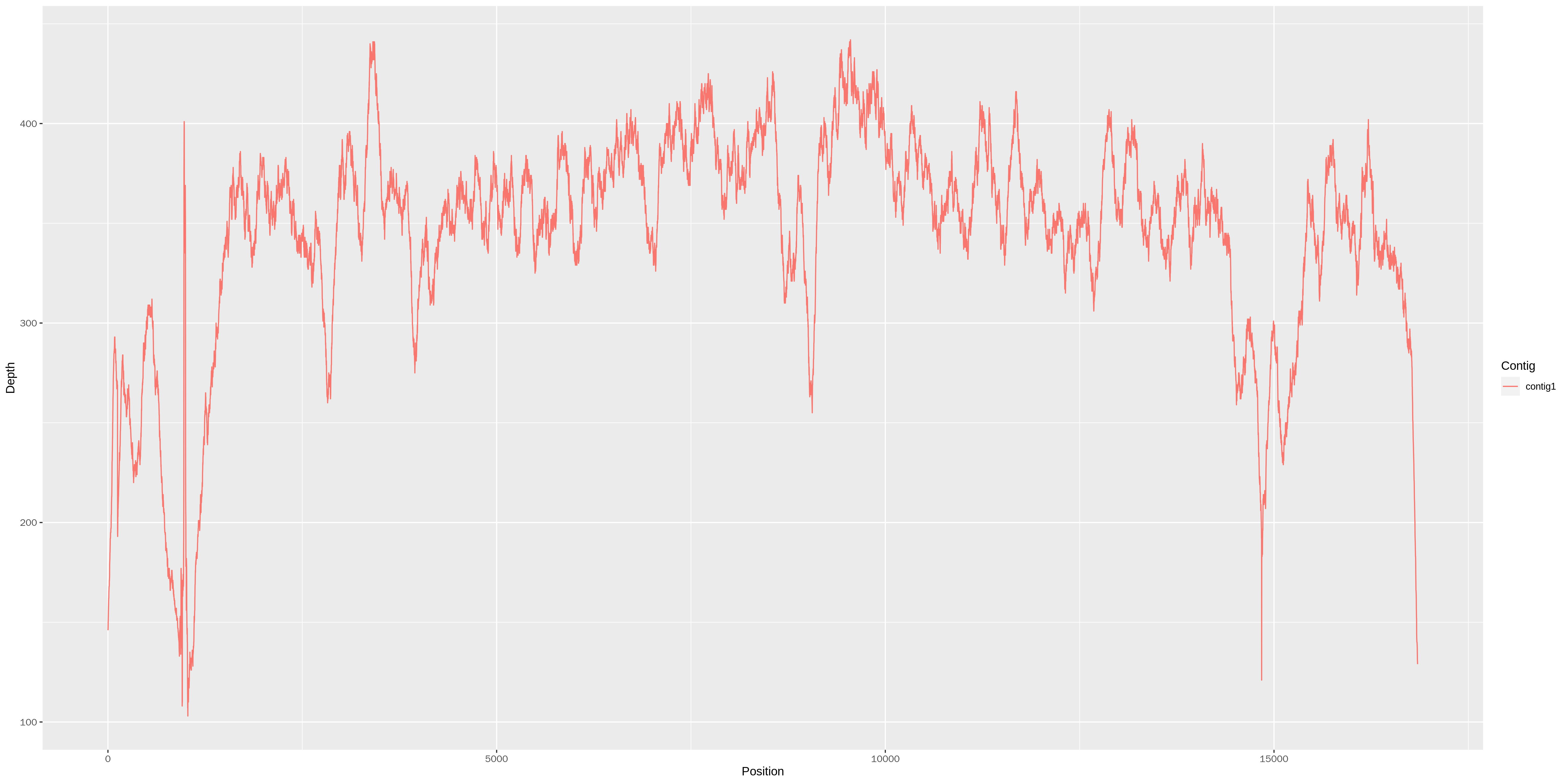


**Supplementary Figure 1.** Coverage depth map of *Chrysemys picta bellii*.


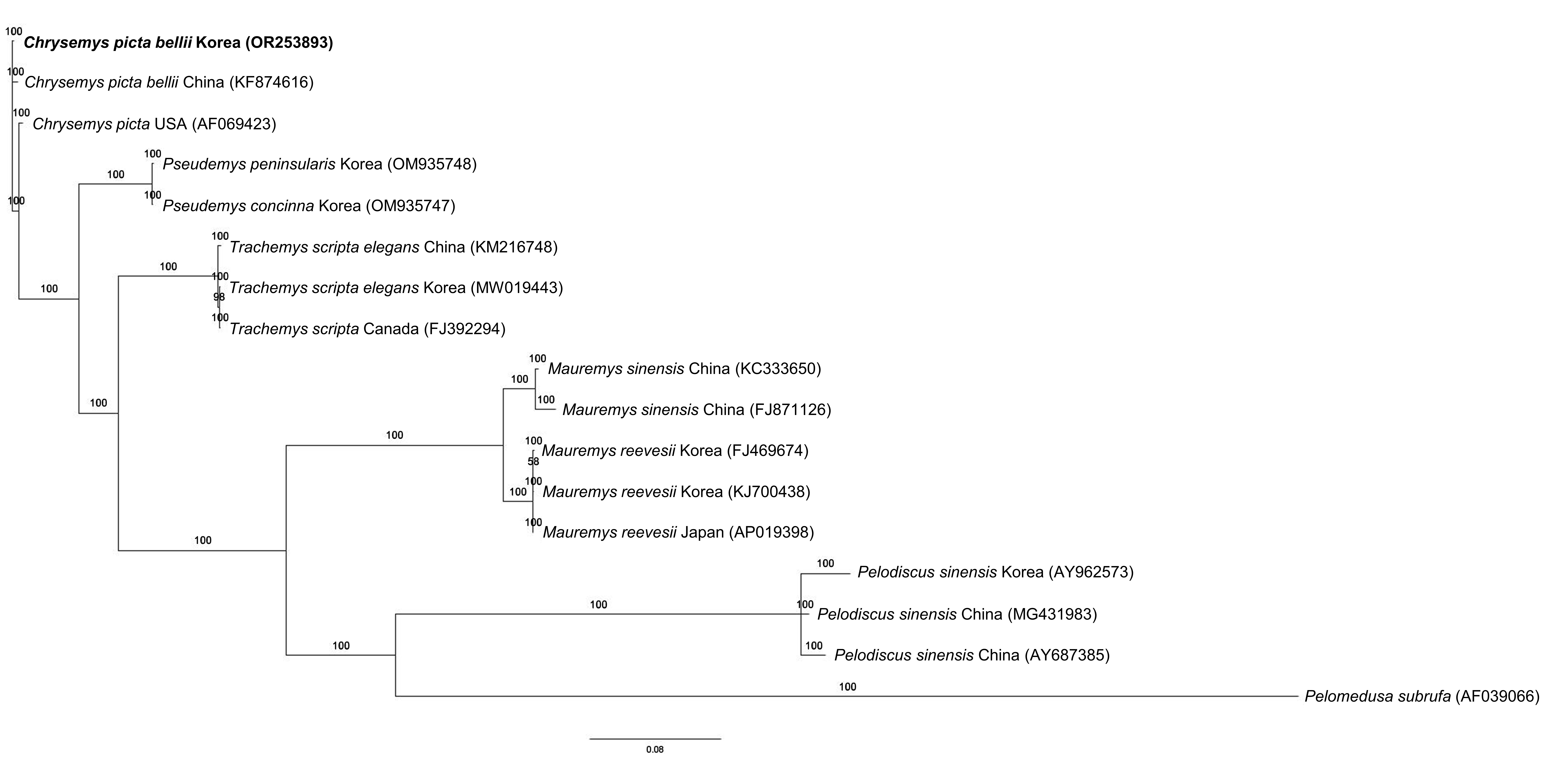


**Supplementary Figure 2.** Phylogenetic tree obtained using Bayesian inference.
